# Electronic patient-reported outcome (ePRO) application for patients with prostate cancer

**DOI:** 10.1371/journal.pone.0289974

**Published:** 2023-08-11

**Authors:** Majid Mohseni, Haleh Ayatollahi, Amir Mohammad Arefpour

**Affiliations:** 1 Department of Health Information Management, School of Health Management and Information Sciences, Iran University of Medical Sciences, Tehran, Iran; 2 Health Management and Economics Research Center, Health Management Research Institute, Iran University of Medical Sciences, Tehran, Iran; 3 Department of Radiation Oncology, School of Medicine, Iran University of Medical Sciences, Tehran, Iran; Lorestan University of Medical Sciences, ISLAMIC REPUBLIC OF IRAN

## Abstract

**Introduction:**

Cancer patients experience different complications and outcomes during or after medical treatments. Electronic reporting of the outcomes by patients is a solution that facilitates communication with physicians and improve patient health status. The aim of this study was to develop a smartphone-based application for electronic reporting of outcomes by patients with prostate cancer.

**Methods:**

The present research was conducted in 2021 in two phases. In the first phase, initially, users’ requirements were identified based on reviewing the related literature, existing applications, and guidelines. Then, a questionnaire was designed and the specialists’ opinions about the users’ requirements were investigated. The specialties included urologists, hemato-oncologists, uro-oncologists, and radiotherapists (n = 15). In the second phase, the application was designed, and patients with prostate cancer (n = 21) and specialists (n = 10) evaluated it using the post-study system usability questionnaire (PSSUQ). Data were analyzed using descriptive statistics.

**Results:**

The findings of the first phase of the research showed that out of 108 data elements and functions proposed for the application, 91 items were found essential by the specialists. Data elements were categorized into the patient data, general complications of prostate cancer and side effects of drug therapy, surgery, chemotherapy, cryotherapy, radiation therapy, and hormone therapy. Necessary functions for the application included presenting a patient care summary, communication between the patient and the specialist, free text explanation for complications and sides effects, generating reports, reminder and alert, completing quality of life questionnaire, and calculating the score for the questionnaire. In the second phase of the research, the application was developed and evaluated. The mean value for user satisfaction was (5.95 ± 0.55) out of 7.

**Conclusion:**

The developed application can help to accelerate communication with the specialists. It can improve quality of care, reduce unnecessary treatment visits and side effects, and improve timely data collection for a variety of research purposes. However, further research on the cost-effectiveness and usefulness of the collected data is recommended.

## Introduction

Prostate cancer is the fourth most common cancer and the eighth leading cause of death in the world [1]. However, less than 20% of men with prostate cancer die from the disease, and 93% of them survive for at least five years after diagnosis [2]. The most important risk factors associated with prostate cancer are age, race, place of residence, family history, and genetic variation. Other risk factors, such as diet, obesity, smoking, exposure to chemicals, prostate inflammation, sexually transmitted infections, and vasectomy can also increase the risk of developing the disease [3]. Treatment of prostate cancer includes a variety of methods, such as drug therapy, surgery, hormone therapy, radiation therapy, cryotherapy, etc. [4].

Most cancer patients experience various symptoms, complications, and side effects. Therefore, facilitating communication between patients and their specialists is important for supporting them [5]. Some studies showed that the use of smartphones has been very useful in monitoring chronic diseases and related outcomes, such as cancer [6–9]. Patients can use mobile-based applications (mhealth technology) to constantly report the therapeutic indications and side effects to their healthcare providers. Continuous communication with the healthcare providers will improve patient’s health condition over time and prevent the recurrence of dangerous outcomes [10]. These data can also be used to implement appropriate care plans, reimbursement strategies, and health policies [11]. Evidence shows that mhealth is effective in supporting patient participation in care, early detection of problems, and maintaining patient safety, and improving individual care [5]. In addition, it seems that patients prefer to use mhealth technology rather than paper forms [12].

One of the systems developed to facilitate communication between patients and specialists is electronic patient-reported outcome (ePRO) system [12]. In this system, a report about the patient health condition can be provided directly by the patient without any interpretation. This report improves clinical workflow as well as symptoms and complications management. The focus of these systems has been mainly on the follow-up care, chemotherapy monitoring [12], monitoring cancer patients’ symptoms [13], radiation therapy monitoring [7, 14], and providing supportive care in radiation therapy [15].

Bennett et al. reviewed the applications of ePRO systems in oncology and found that these systems support a variety of clinical activities, including chemotherapy, radiation therapy, postoperative monitoring, symptom management during palliative care, and home care. They also increase the efficiency and quality of care; and facilitate better communication with the specialists [16]. In general, ePRO provides a more comprehensive and meaningful clinical insight in terms of screening, diagnosis, and response to treatment [17, 18]. An example of ePRO is eSMART application, which was designed to manage symptoms in a European multicenter study, and to assist patients with breast and colon cancer who were under chemotherapy [19]. In another study, a patient remote intervention and symptom management system (PRISMS) was design and used as an intervention for patients with malignant cancers [20].

Regarding prostate cancer, several studies have been conducted on the use of the electronic patient-reported outcomes [5, 14, 21–23]. In a study with the aim of examining the feasibility of the electronic patient-reported outcome, the quality of life of patients with prostate cancer was assessed using validated questionnaires [23]. Paterson et al. also conducted a prospective longitudinal study on the use of the electronic report of outcomes by prostate cancer survivors. The participants complained about a lack of social support during cancer treatment [22]. In a study conducted by Holch et al., the results showed that patients with prostate cancer needed help with severe complications of treatment, disease management, support, and telephone counseling [14]. However, it seems that a limited number of studies have been conducted on the use of smartphone-based applications for electronic reported outcomes by patients with prostate cancer [5, 7]. Other studies have often focused on the feasibility, effectiveness, and evaluation of the electronic patient-reported outcome for different types of cancer [5, 12, 24, 25], and the side effects of radiation therapy [7, 14, 15]. The designed programs were mainly web-based [26–29] or were provided via a specific portal [30] and created for being used in the clinics and healthcare centers [11]. Therefore, the present study aimed to develop a smartphone-based application for electronic reporting of outcomes by patients with prostate cancer.

## Methods

The present research was conducted in two phases in 2021. In the first phase, the data elements and functions required for the application were identified quantitatively using a questionnaire. In the second phase, using the data obtained from the first phase, the application was developed and evaluated. Before conducting the research, the ethical approval code (IR.IUMS.REC.1399.679) was obtained from the national committee for ethics in biomedical research.

### Phase 1: Determining the data elements and functions required for the application

In the first phase of the study, the potential participants were 30 specialists including urologists (n = 14), hemato-oncologists (n = 7), uro-oncologists (n = 4), and radiotherapists (n = 5) who worked in three different teaching hospitals affiliated to one of the medical universities. No sampling method was used and all of the specialists who had the experience of treating patients with prostate cancer were invited to complete a questionnaire in January-February 2021. The research questionnaire was designed to determine the data elements and functions required for the application based on the literature review, existing applications, and guidelines of the US Centers for Disease Control, National Cancer Institute, and the American Cancer Society [2, 17, 18, 20, 23, 28, 29, 31]. In order to search the literature, databases like PubMed, Scopus, and web of Science were searched using keywords such as electronic patient-reported outcome (ePRO), prostate cancer, health related-quality of life, etc.

This questionnaire included the necessary data elements (patient data (21 items), general complications of prostate cancer (18 items), side effects of drug therapy (9 items), surgery (15 items), chemotherapy (10 items), cryotherapy (3 items), radiation therapy (8 items), hormone therapy (16 items) and functional requirements of the application (8 items). The questionnaire had two choices; namely, “necessary” and "unnecessary" for each item. Those items which were found necessary by at least 60% of the respondents were included in the smartphone-based application. The validity of the questionnaire was assessed by three urologists, and the reliability was calculated using Kuder-Richardson formula-20 (KR-20 = 0.91). All participants signed a written consent form before completing the questionnaires. Data were analyzed using descriptive statistics and Statistical Package for the Social Sciences (SPSS) version 26 was used to facilitate data analysis.

### Phase 2: Designing and evaluating the application

A smartphone-based application for electronic patient-reported outcome was developed using Android Studio environment and Basic 4 Android programming language, and its usability was evaluated using the post-study system usability questionnaire (PSSUQ). In this phase, 21 patients and 10 specialists used the application and participated in the evaluation study in November-December 2021.

After two weeks of using the application, patients and specialists were asked to complete the PSSUQ. It is a 16-item standardized questionnaire which is widely used to measure users’ perceived satisfaction with a website, software, system or product at the end of a study. Although there are various questionnaires to measure usability, a number of factors such as the general content, advantages and disadvantages, coverage of usability aspects, and psychometric support need to be considered when choosing the most appropriate measure(s) to use. Other factors that may affect selecting a questionnaire include financial resources, time, usefulness, and difficulty of measuring [32]. Therefore, we decided to use the PSSUQ which was more appropriate for our research.

The PSSUQ was a seven-point Likert scale (strongly disagree (1) to strongly agree (7)) questionnaire and included 16 questions in three sections: system quality (6 questions), information quality (6 questions), and system interface quality (4 questions). Similar to Schnall et al., the higher scores indicated the higher user satisfaction [33]. All participants signed a written consent form before completing the questionnaires. The data were analyzed using descriptive statistics. The reliability of the questionnaire was calculated using Cronbach’s alpha coefficient (α = 0.83).

## Results

The research findings are presented in three sections: data elements and functions required for the application, the application development, and its usability evaluation.

### Data elements and functions required for the application

In the first phase of the study, 15 specialists out of 30 (50%) completed the questionnaire. All participants were men and the highest frequency (n = 6, 40%) was related to the age group of 40–45 years. The mean age of the participants was 44.90 ± 3.51 years. As mentioned earlier, the questionnaire consisted of nine sections including patient data, general complications of prostate cancer, side effects of drug therapy, surgery, chemotherapy, cryotherapy, radiation therapy, hormone therapy and functional requirements of the application. The results showed that 91 out of 108 items of the questionnaire were found “necessary” by at least 60% of the participants and were included in the application. Table 1 shows the participants’ responses about the necessary patient data which should be included in the application. In this section, father’s name and history of vasectomy were found unnecessary and removed from the final list of necessary data for the application.

**Table 1:** Participants’ responses about the necessary patient data.

NO	Patient data	Necessary		Unnecessary		Missing data	
		Frequency	Percent	Frequency	Percent	Frequency	Percent
1	Name	9	60	4	7.26	2	3.13
2	Surname	9	60	4	7.26	2	.313
3	Father’s name	6	40	7	7.46	2	3.13
4	National ID	10	7.66	3	20	2	3.13
5	Job	11	3.73	2	3.13	2	3.13
6	Race	10	7.66	5	3.33	0	0
7	Education	9	60	6	40	0	0
8	Marital status	10	7.66	5	3.33	0	0
9	Age	15	100	0	0	0	0
10	City of residence	10	7.66	3	20	2	3.13
11	Contact number	12	80	1	7.6	2	3.13
12	Family history of cancer	12	80	3	20	0	0
13	Height	11	3.73	4	7.26	0	0
14	Weight	14	3.93	1	7.6	0	0
15	Smoking status	14	3.93	1	7.6	0	0
16	History of Vasectomy	7	7.46	7	7.46	1	7.6
17	Body Mass index (BMI)	14	3.93	1	7.6	0	0
18	History of diabetes	12	80	2	3.13	1	7.6
19	Duration of prostate cancer	15	100	0	0	0	0
20	Secondary cancer	15	100	0	0	0	0
21	Type of treatment	15	100	0	0	0	0

In terms of general complications of prostate cancer, the results showed that memory loss and confusion, mouth and throat disorders, skin and hair disorders, and sleep disorders were not found necessary to be considered in the application (Table 2).

**Table 2:** Participants’ responses about the general complications of prostate cancer.

No	General complications of prostate cancer	Necessary		Unnecessary		Missing data	
		Frequency	Percent	Frequency	Percent	Frequency	Percent
22	Low back pain	12	80	2	1	1	6.7
23	Anemia	14	93.3	1	0	0	0
24	Loss of appetite	12	80	2	1	1	6.7
25	Thrombocytopenia	9	60	6	0	0	0
26	Digestive system disorders	11	73.3	3	1	1	6.7
27	Edema	15	100	0	0	0	0
28	Infection	12	80	3	0	0	0
29	Lymph node edema	12	80	3	0	0	0
30	Memory loss and confusion	5	33.3	10	0	0	0
31	Mouth and throat disorders	6	40	9	0	0	0
32	Nervous system disorders	9	60	6	0	0	0
33	Pain	15	100	0	0	0	0
34	Sexual problems	11	73.3	4	26.7	0	0
35	Skin and hair disorders	5	33.3	10	7.66	0	0
36	Sleep disorders	7	46.7	7	.746	1	.76
37	Urinary system disorders	15	100	0	0	0	0
38	Mental disorders	12	80	3	20	0	0
39	Drug addiction	13	86.7	1	7.6	1	.76

Regarding the side effects of drug therapy, nausea and headache were excluded (Table 3). In terms of the surgical side effects, problems after anesthesia, nerve and vascular damages as well as hernia, and regarding the side effects of chemotherapy, hair loss were removed from the final list (Table 4).

**Table 3:** Participants’ responses about the side effects of drug therapy.

NO	Side effects of drug therapy	Necessary		Unnecessary		Missing data	
		Frequency	Percent	Frequency	Percent	Frequency	Percent
40	Sexual problems	13	7.86	1	7.6	1	.76
41	Nausea	7	7.46	6	40	2	3.13
42	Headache	6	40	7	.746	2	3.13
43	Hot flashes	13	7.86	1	.76	1	7.6
44	Low back pain	13	7.86	2	3.13	0	0
45	Osteoporosis	13	7.86	1	7.6	1	7.6
46	Hypertension	11	3.73	4	.726	0	0
47	Musculoskeletal pain and arthritis	11	.373	4	7.26	0	0
48	Mental disorders	11	.373	4	.726	0	0

**Table 4:** Participants’ responses about the side effects of surgery, chemotherapy, and cryotherapy.

NO	Side effects		Necessary		Unnecessary	
			Frequency	Percent	Frequency	Percent
49	**Surgery**	Fecal incontinence	10	100	0	0
50		Leakage of urine from the anus	7	86.7	2	13.3
51		Sexual problems	13	93.3	1	6.7
52		Urinary system disorders	12	93.3	1	6.7
53		Fatigue	13	60	6	40
54		Urinary incontinence	10	100	0	0
55		Problems after anesthesia (headache, bruising, itching, etc.)	12	46.7	8	53.3
56		Heart attack	15	66.7	5	33.3
57		Stroke	1	60	6	40
58		Deep vein thrombosis	7	100	0	0
59		Nerve and vascular damages	13	53.3	7	46.7
60		Infection	12	80	3	20
61		Bleeding	13	86.7	2	13.3
62		Hernia	10	53.3	7	46.7
63		Lymph node edema	12	93.3	1	6.7
64	**Chemotherapy**	Digestive system disorders	12	80	3	20
65		Fatigue	9	60	6	40
66		Mouth ulcers	10	66.7	5	33.3
67		Hair loss	7	46.7	8	53.3
68		Leucopenia	13	86.7	2	13.3
69		Easy bleeding and bruising	12	80	3	20
70		Thrombocytopenia	13	86.7	2	13.3
71		Weakness	10	66.7	5	33.3
72		Infection	12	80	3	20
73		Anemia	15	100	0	0
74	**Cryotherapy**	Sexual problems	13	.786	2	3.13
75		Urinary incontinence	14	3.93	1	7.6
76		Leakage of urine from the anus	13	7.86	2	3.13

Some side effects of radiation therapy including pain and skin disorders, and some side effects of hormone therapy, namely, digestive system disorder, fatigue, confusion and pain were also exuded from the final list (Table 5).

**Table 5:** Participants’ responses about the side effects of radiation and hormone therapy.

No	Side effects		Necessary		Unnecessary		Missing data	
			Frequency	Percent	Frequency	Percent	Frequency	Percent
77	**Radiation therapy**	Sexual problems	14	3.93	1	7.6	0	0
78		Fatigue	9	60	5	3.33	1	7.6
79		Urinary system disorders	14	3.93	1	7.6	0	0
80		Pain	7	7.46	7	7,46	1	7.6
81		Digestive system disorders	10	7.66	4	7,26	1	7/6
82		Skin disorders	5	3.33	8	3.53	2	3.13
83		Edema	13	7.86	1	7.6	1	7.6
84		Lymph node edema	13	7.86	1	7.6	1	7.6
85	**Hormone therapy**	Breast growth and sensitivity	14	.393	1	7.6	0	0
86		Sexual problems	14	3.93	1	7.6	0	0
87		Digestive system disorders	8	3.53	7	7.46	0	0
88		Cardiovascular disorders	12	80	3	20	0	0
89		Osteoporosis	10	7.66	5	3.33	0	0
90		Weight gain	9	60	6	40	0	0
91		Hot flashes	14	3.93	1	7.6	0	0
92		Fatigue	8	.353	5	3.33	2	3.13
93		Loss of muscles	11	3.73	3	20	1	7.6
94		Confusion	6	40	9	60	0	0
95		Heart attack	10	7.66	5	3.33	0	0
96		Pain	7	.746	8	3.53	0	0
97		Anemia	10	7.66	4	7.26	1	7.6
98		Cholesterol disorders	11	3.73	4	7.26	0	0
99		Insulin resistance	9	60	5	3.33	1	7.6
100		Behavioral disorders	9	60	6	40	0	0

Regarding the required functions, as Table 6 shows, all items were found necessary by the respondents.

**Table 6:** Participants’ responses about the required functions in the application.

No	Required functions	Necessary		Unnecessary		Missing data	
		Frequency	Percent	Frequency	Percent	Frequency	Percent
101	Presenting a patient care summary	12	80	2	3.13	1	7.6
102	Sending a message to the physician	13	80	1	7.6	1	7.6
103	Sending a reply to the patient	13	80	1	7.6	1	7.6
104	Free text explanation for complications and sides effects	11	3.73	3	20	1	7.6
105	Generating reports	14	3.93	1	7.6	0	0
106	Reminder and alert	14	3.93	1	7.6	0	0
107	Completing quality o life questionnaire	10	7.66	3	20	2	3.13
108	Calculating the score for the questionnaire	12	80	2	3.13	1	7.6

### Application development

In the second phase of the research, the application was developed based on the findings of the first phase. This application included two separate panels for the specialists and patients.

### Specialist’s panel

**Fig 1:**
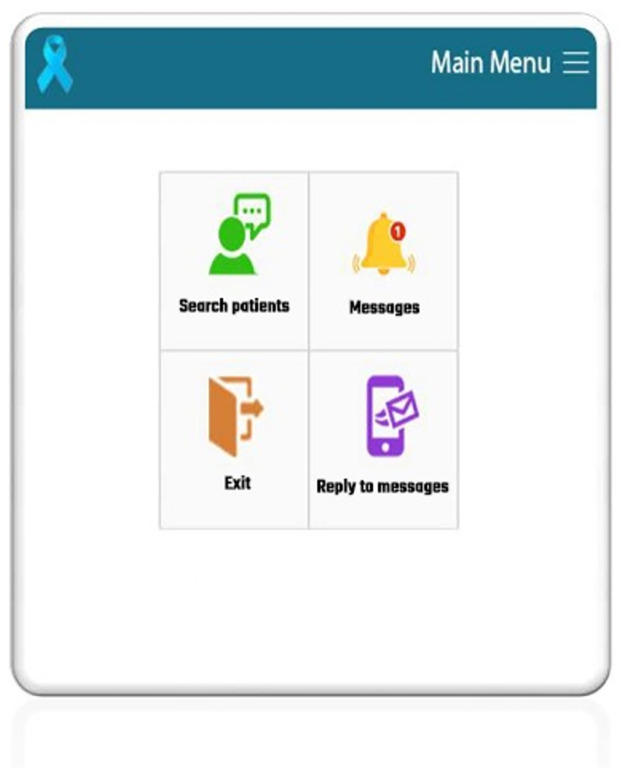
Specialists’ panel in the application.

In the specialist’s panel, the application helped the user to search for patients using the patient’s name, surname, and national ID, to access patient clinical records and outcomes, to see a list of received messages, and to communicate with the patients by answering their queries (Fig 1).

### Patient’s panel

**Fig 2:**
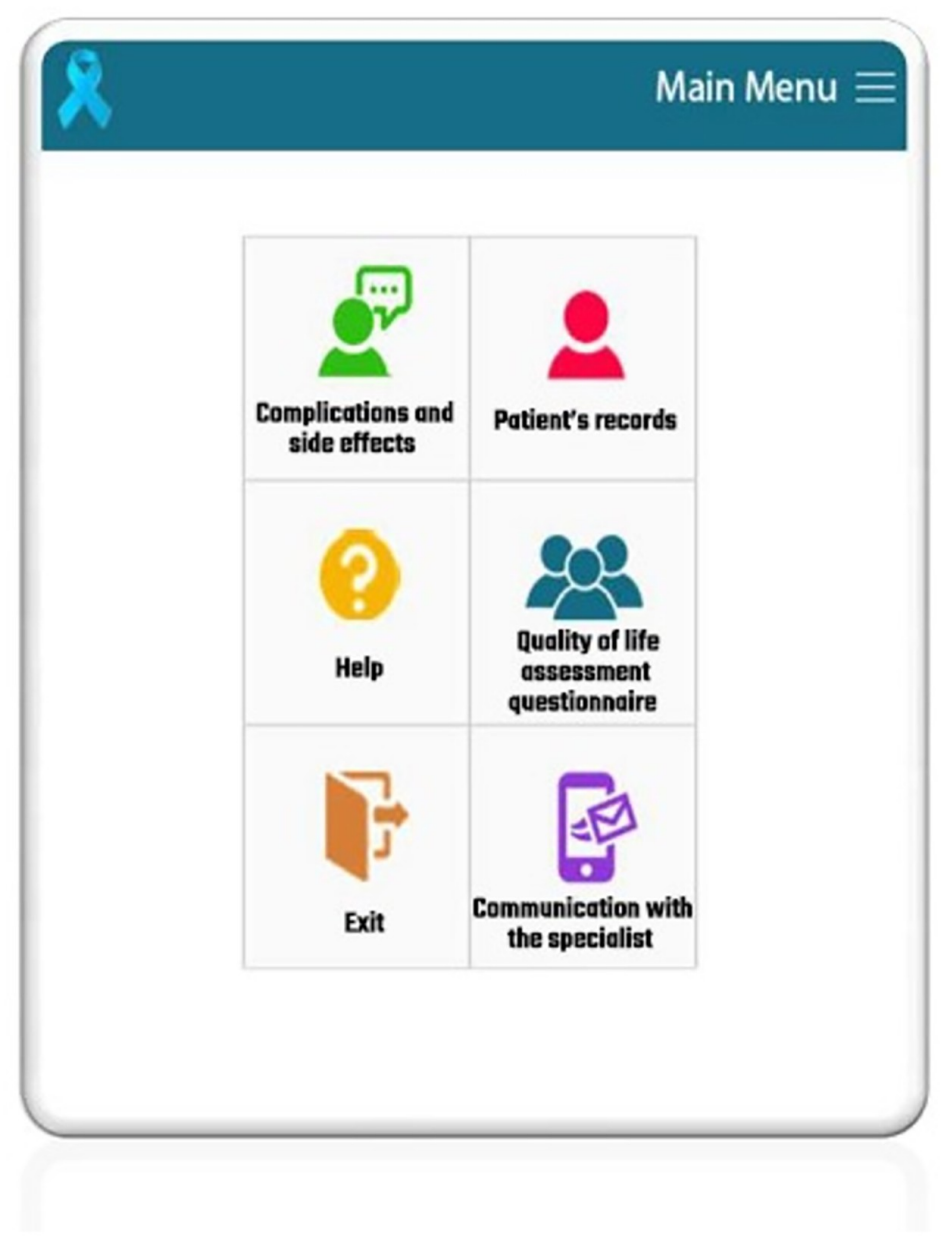
Patient’s panel in the application.

**Fig 3:**
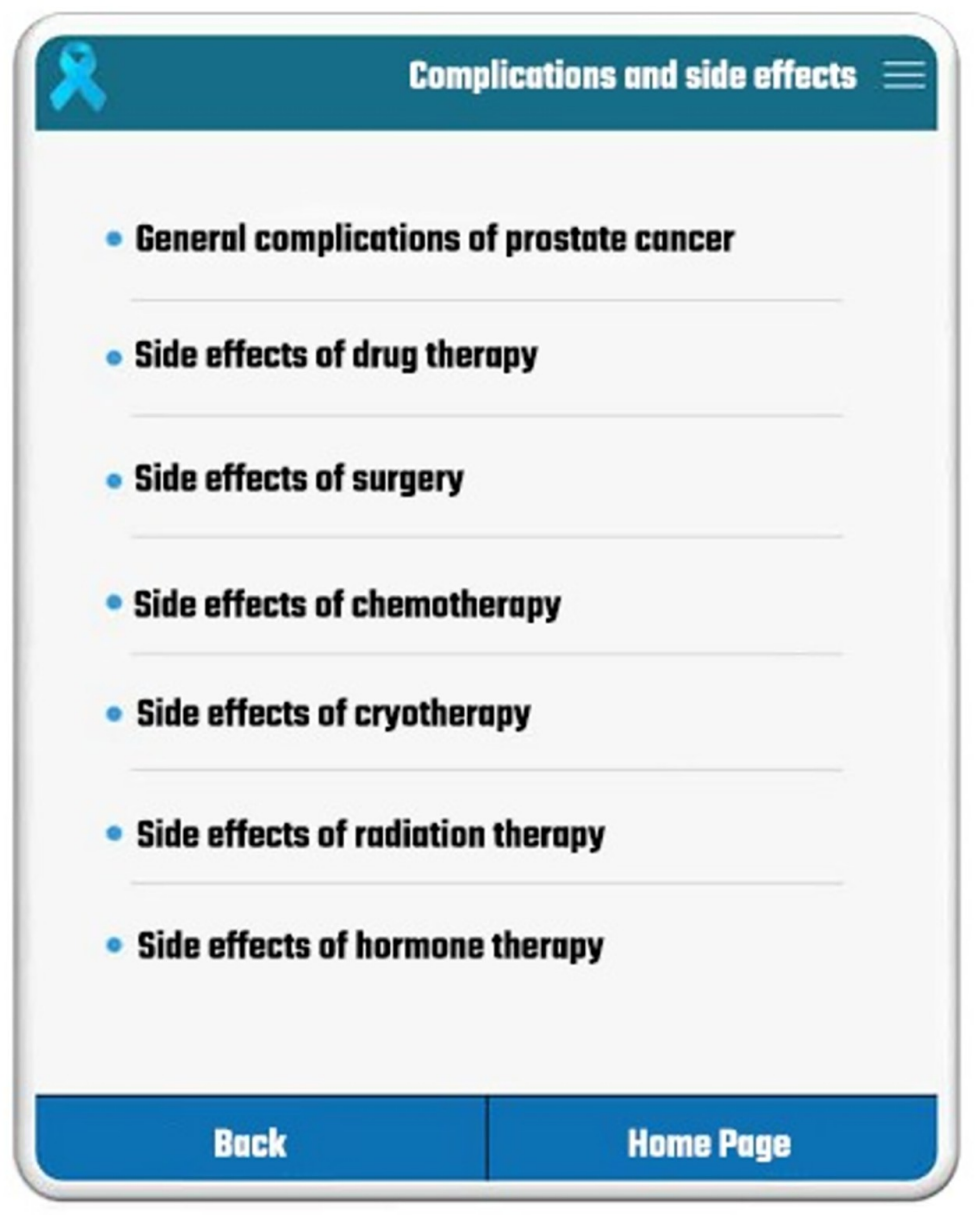
Side effects of cancer treatment procedures.

The patient’s panel of the application included patient’s records including personal and clinical data, and body mass index, general complications of prostate cancer, and side effects of drug therapy, surgery, chemotherapy, cryotherapy, radiation therapy, hormone therapy, quality of life assessment questionnaire, and communication with the specialist (Figs 2 and 3).

In case of any type of side effects, patients could use a dropdown menu to enter yes or no answers, and in case of additional explanations, they could enter more data in the related box. Another part of the patient’s panel devoted to the “quality of life assessment questionnaire”. This questionnaire included various aspects of the patient heath condition (e.g., psychological, emotional, physical aspects, etc.) and helped the specialists to gain a better understanding of the patient’s quality of life. After completing the questionnaire, patients could also see the results and a report about their mental health, general health, physical function, etc. In case of any serious problems or side effects, patients could communicate with their specialist via the application. The specialists were informed about this communication by receiving a Short Message Service (SMS) and could answer the patient by logging into the application and using the related section to reply.

Finally, the usability of the application was evaluated by using the PSSUQ. The participants included patients with prostate cancer and their specialists. The highest frequency of patients (n = 10, 47.6%) aged 66 years and older, and the mean age of patients was 66.10 ±5.53 years. In terms of education, all patients had either high school diploma or were less educated (100%). The most common treatment method received by patients was surgery (n = 12, 57.1%) and the average of cancer care was 28.29±14.09 months for them. Among the specialists, the highest frequency of the participants was men (n = 7, 70%) and 50% of the participants aged 41–45 years old. The specialists’ mean age was 40.89±5.71 years. The application was available to the patients and their specialists for two weeks and then, they were asked to complete the questionnaire. The results of the application evaluation are presented in Table 7.

**Table 7:** Patients’ and specialties’ opinions about the application.

Title	Mean and standard deviation		
	Specialists	Patients	Total
System quality	6.13± 0.50	6.01± 0.47	6.07± 0.48
Information quality	5.77±0.72	5.68±0.40	5.72±0.56
User interface quality	6.00± 0.79	6.15± 0.44	6.07± 0.61
Total	5.97± 0.67	5.92± 0.45	5.95± 0.55

The results showed that the mean values for system quality (6.07 ± 0.48) and user interface quality (6.07 ± 0.61) were higher than the mean value for information quality for both groups of the participants. The total mean value and standard deviation for the application was (5.95 ± 0.55) out of 7.

## Discussion

In the present study, a smartphone-based application was developed for electronic patient-reporting of the complications and side effects of treatment procedures of prostate cancer. In the first phase of the study, data elements and functions required for the application were identified based on the specialists’ perspectives. In the second phase of the research, the application was developed and its usability was evaluated by patients and their specialists. The results showed that overall; both groups were satisfied with the application.

An electronic patient-reported outcome application provides a comprehensive picture of patient’s health status and quality of life. It is available at any time and any place, and facilitates patient assessment at home [12]. Moreover, electronic data collection has many practical advantages over the paper-based methods. For example, data collection, storage, and analysis get easier, the validity of data increases, and more appropriate care plans can be provided for patients [27].

Reporting complications and outcomes by patients also helps to improve care and prevents unnecessary visits. This process improves patient safety and quality of life, reduces costs, and improves physician-patient communication. Such a system supports online reporting and tracking patients’ symptoms, too. Furthermore, a more complete patient record can be created and integrated with other patient data sources [28].

According to the literature, previous studies have mainly focused on electronic reporting of the outcomes and side effects of radiation therapy, surgery, and chemotherapy in cancer patients. For instance, Hauth et al. focused on the side effects of radiation therapy and highlighted their impact on the patients’ quality of life [29]. The Advanced Symptom Management System (ASyMS) was a mobile-based application developed to assess symptoms of patients with prostate cancer during chemotherapy [34]. Another system was Symptom Tracking and Reporting (STAR) software used in complete prostatectomy surgery and stored the symptoms reported by the patient during chemotherapy and after surgery. In this study, a web-based questionnaire was completed by patients 3, 6, 9, 12, 18, 24, 36, and 48 months after surgery, and questionnaire responses were automatically added to their electronic medical record [35]. The TellUs software allowed patients with prostate cancer and their caregivers to communicate with their healthcare providers during cancer treatment. It helped with easy and immediate sharing of patients’ queries, and improved their quality of life [36]. However, in the current study, different types of treatment procedures and their side effects were investigated and added to the application to be able to present a more complete picture of patient health condition.

Similar to the current study, Tran et al. designed the “Strength Through Insight” application to collect electronic patient-reported outcomes from patients with prostate cancer. Using this application, patients’ quality of life was assessed by the quality of life questionnaires, Expanded Prostate Cancer Index Composite questionnaire (EPIC), and Functional Assessment of Cancer Therapy Advanced Prostate Symptom Index (FACT-P Symptom Index) [23]. In another study, a web-based application called “Prometheus” was created for patients who received radiation therapy. The results showed that this system had a lot of potentials to improve the care process by providing complete documents of the patient symptoms and outcomes [29]. “Patient Viewpoint” collected electronic patient-reported outcomes during a visit by physicians. The patient’s outcome score was automatically added to the patient’s electronic health records, and displayed graphically and numerically to the clinicians. Patients could visit the website at any time to see the changes in the outcome score over time [30]. Similarly, in the current study, apart from reporting complications and side effects, patients could complete a quality of life questionnaire and its score was visible to the patient and the specialist to compare its changes overtime.

In Patient Care Monitor (PCM) software, patients used a tablet to complete a questionnaire during the visit. The questionnaire consisted of 80 questions related to the general and physical symptoms, functions, surgical complications, treatment side effects, anxiety, and disappointment and could be used in various types of cancers, including genitourinary tract, lung, skin/sarcoma, and breast cancer [31, 34]. However, in the current study, a smartphone-based application was specifically developed for patients with prostate cancer, and the users’ could easily install it on their smartphones. Moreover, patients could complete any parts of the application which was relevant to their health condition or the type treatment that they received.

According to the literature, few studies evaluated their developed systems. However, those in which systems were evaluated, their findings showed that the system led to improve quality of life and patient satisfaction [25], and most patients had a positive attitude towards using these systems in the future [37, 38]. Similar to other studies, the application developed in the current study was evaluated by patients and their specialists. In their opinion, the application was simple and easy to use for different educational background, could be used to improve patient care, and could reduce complications and treatment side effects.

Considering the importance of telemonitoring services for managing cancer patients, it seems that the application develop in the current study can provide both patients and healthcare providers with the potential benefits of ePRO. The application can help to identify patients that require further health assessment and potential therapeutic changes at the very early stages of treatment. However, further research is needed to fully understand its potential impact on patient outcomes, and integrate collected data into the rest of patient’s medical records.

### Research limitations

In this research, although we tried to include as many as complications and side effects of cancer treatment procedures for patients with prostate cancer, the research had some limitations. One of the limitations of the present study might be related to the limited number of the participants in both phases of research. In fact, this research was conducted during the Covd-19 pandemic when most of the specialists were busy and their workload was affected by the pandemic. Moreover, patients might be reluctant to participate in the study due to their health conditions. Secondly, in the first phase of the research, we only invited specialists to determine what the patients may need to report. As the specialists were more aware of the possible outcomes of cancer treatments, and electronic patient-reported outcome data can be used for research purposes and we preferred to complete this phase without involving cancer patients. However, in the future patients can be involved more to examine the effectiveness of the application. Thirdly, only one questionnaire was used to evaluate the application. It seems that using other quantitative and qualitative evaluation methods can help to gain a better understanding about the strengths and limitations of the application.

## Conclusion

In this study, a mobile-based application was developed for electronic reporting of outcomes and treatment side effects by patients with prostate cancer. The results showed that both patients and their specialists were satisfied with the application at a high level. It seems that patients can easily report any outcomes or treatment side effects by using this application, and can contact their specialist at the point of need. The collected data can be used for conducting clinical research, improving patients’ awareness and quality of life, reducing unnecessary clinical visits, and facilitating patient access to healthcare services. However, further research on the cost-effectiveness of the application and integrating collected data into the rest of patient medical records is recommended.

## Supporting information

S1 Checklist STROBE statement—Checklist of items that should be included in reports of *cross-sectional studies*. (DOCX)

## Abbreviations

ASyMS: Advanced Symptom Management System
EPIC: Expanded Prostate Cancer Index Composite
ePRO: Electronic Patient-Reported Outcome
FACT-P Symptom Index: Functional Assessment of Cancer Therapy- Advanced Prostate Symptom Index
PCM: Patient Care Monitor
PRISMS: Patient Remote Intervention and Symptom Management System
PSSUQ: Post-Study System Usability Questionnaire
SMS: Short Message Service
SPSS: Statistical Package for the Social Sciences
STAR: Symptom Tracking and Reporting
